# Malrotation with mid-gut volvulus and partial small bowel peritoneal membrane encapsulation

**DOI:** 10.4102/sajr.v28i1.2936

**Published:** 2024-08-28

**Authors:** Ryan Frerichs, Tanusha Sewchuran

**Affiliations:** 1 Department of Radiology, Grey’s Hospital, Pietermaritzburg, South Africa

**Keywords:** malrotation, bowel obstruction, volvulus, congenital peritoneal encapsulation, upper gastrointestinal fluoroscopy

## Abstract

Urgent investigation is crucial for infants with bilious vomiting, potentially indicating bowel obstruction. Upper gastrointestinal fluoroscopy aids diagnosis, but is not without its challenges. This case report describes a rare case of neonatal intestinal malrotation and mid-gut volvulus with an additional complication of congenital peritoneal encapsulation.

**Contribution:**

This case study offers insights into associated diagnostic challenges and underscores the value of utilising fluoroscopy in diagnosing complex gastrointestinal conditions.

## Introduction

The urgent investigation of bilious vomiting in infants is crucial, as it may indicate partial or complete bowel obstruction distal to the ampulla of Vater.^1^ While the causes of bowel obstruction are numerous, the preferred initial imaging method for diagnosis is upper gastrointestinal (UGI) fluoroscopy using water-soluble contrast medium.^1,2^ Special consideration should be taken to observe for signs of intestinal malrotation and related secondary complications.^1^

Intestinal malrotation, found in approximately 1 in 500 births, is a congenital anomaly involving abnormal positioning of the bowel within the peritoneal cavity.^3^ It occurs because of incomplete rotation of the intestines during foetal development and failure of bowel fixation.^3,4^ Normally, between the 5th and 12th weeks of gestation, the bowel herniates into the umbilical cord and undergoes counter-clockwise rotation around the superior mesenteric artery before returning to its place in the peritoneal cavity.^5^ As a result, the duodenojejunal (DJ) junction, held in position by the ligament of Treitz, is located in the left upper quadrant, with the mesenteric fixation extending broadly to the caecum in the right lower quadrant.^5^

Although varying degrees of malrotation may occur, classically, malrotation involves an abnormal arrangement of both the small and large bowel, with the large intestine lying on the left and the small intestine on the right.^4,6^ The DJ junction is positioned lower and to the right of its usual location, while the caecum tends to be elevated and positioned more towards the midline.^5^ The mesenteric attachment of the mid-gut, particularly from the DJ junction to the caecum, is unusually short, fixated by abnormal coloduodenal peritoneal bands (known as Ladd’s bands) crossing from the mal-positioned caecum to the lateral peritoneal gutter over the duodenum, causing obstruction.^7,8^ There can be absence of fixation of sections of the bowel, predisposing to volvulus.^6^ Consequently, there is a predisposition for the gut to rotate counter-clockwise around the superior mesenteric artery and vein, increasing the risk of bowel obstruction, acute or chronic volvulus and bowel necrosis.^6^ These complications can be life-threatening, with a mortality rate estimated at approximately 3%.^9^

In this case study, we discuss a neonate who presented with bilious vomiting, prompting investigation to assess for potential bowel obstruction. The findings of this study reveal an atypical manifestation of malrotation and mid-gut volvulus, with a concurrent, rare finding of congenital peritoneal encapsulation (CPE).

## Ethical considerations

All procedures performed in this study were in accordance with the ethical standards of the institutional research committee and with the 1964 Helsinki Declaration and its later amendments or comparable ethical standards. Written informed consent was obtained from all individual participants involved in the study via parental consent, which included the use of all images and clinical data.

## Patient Presentation

A 6-day-old neonate was admitted with bilious vomiting, raising suspicion of gastrointestinal obstruction. Upon examination, the abdomen was soft, non-tender and undistended. Nasogastric aspirates confirmed the presence of bile.

**FIGURE 1:**
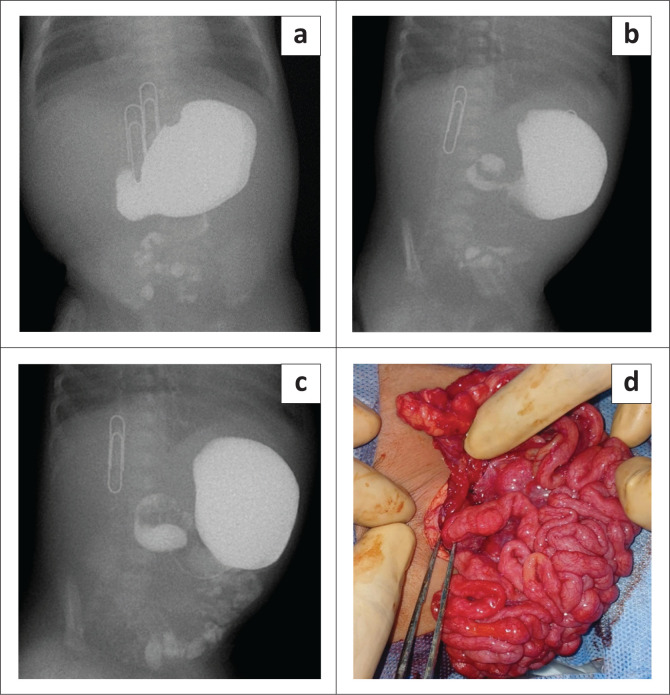
(a) An anterior-posterior projection film of an upper gastrointestinal series done under fluoroscopy for suspected bowel obstruction. The paperclips indicate the midline. There is absence of a normal descending C-loop of duodenum with scanty contrast into the small bowel. The small bowel loops appear to be centrally located. (b) A right anterior oblique projection film of the same patient demonstrates an elevated pylorus with the duodenum oriented postero-superiorly. (c) A right anterior oblique projection further shows that the duodenum appears to be oriented postero-superiorly with dilatation of the first segment. (d) Intra-operative photo of a 6-day-old neonate who underwent an exploratory laparotomy for intestinal obstruction. The forceps is indicating an intraperitoneal membrane encasing the duodenum creating the impression of a duodenal cocoon.

Initial investigations included an abdominal radiograph, which demonstrated signs of proximal bowel obstruction. Consequently, a water-soluble contrast swallow was requested and performed under fluoroscopy. During the procedure, 40 mL of Omnipaque was administered in incremental doses through the nasogastric tube. No abnormalities were observed in the oesophagus, and no reflux detected. The stomach appeared adequately distended in the left upper quadrant, with no delay in gastric emptying on immediate imaging. However, there was scanty contrast passing through the pyloric sphincter into the small bowel, with the pylorus appearing elevated and the duodenum oriented postero-superiorly (Figure 1a–c). This orientation led us to believe that the DJ flexure was on the right and more superior than expected, possibly obscured by the contrast filled stomach. There was evidence of persistent dilatation of D1, with concerns for segmental narrowing in the D2 segment, which may have represented a short segment stricture or ‘corkscrew sign’. The assessment concluded that the abnormal orientation of the gastric pylorus and duodenum might suggest non-classic gut malrotation, and the elevated pylorus and proximal duodenum along with narrowing at D2, raised concern for an annular pancreas or antral web.

Following consultation with the paediatric surgery department and review of the fluoroscopy images, the clinical presentation of bilious vomiting coupled with abnormal orientation of the duodenal c-loop led to the decision to perform an exploratory laparotomy. During laparotomy, the presence of malrotation with mid-gut volvulus, with two twists, was confirmed. Thickened Ladd’s bands were found as well as an accessory intraperitoneal membrane encasing the duodenum creating a cocoon-like structure, which was later diagnosed as CPE (Figure 1d). Interestingly, a concurrent heterotopic pancreas was identified adjacent to D4 of the duodenum, while the DJ junction was found to the right of the spine.

Prompt surgical intervention ensued, including devolving of the bowel in an anticlockwise direction and performing a Ladd’s procedure to rectify the malrotation. Additionally, the heterotopic pancreas was carefully assessed, with no immediate surgical intervention deemed necessary. Postoperative recovery was uneventful, with the patient commencing oral feeds on day 4 and achieving full feeds by day 7 before discharge. Subsequent clinical follow-ups confirmed the patient’s satisfactory progress, with no further complications attributable to the condition or the surgery.

The multidisciplinary approach, incorporating prompt radiological investigation, surgical expertise, neonatal care and parental support, allowed for full recovery and favourable outcomes.

## Discussion

Effective communication between the examining surgeon and the radiologist is pivotal for streamlining the most appropriate imaging modality and discussing potential differentials when the diagnosis is uncertain. In this particular case, intestinal malrotation emerged as the primary consideration, with an atypical appearance of the DJ junction anatomy and a postero-superior orientation of the proximal duodenum. Although a suspected short segment corkscrew appearance indicative of volvulus was observed (Figure 1b), the impression of a narrow stricture might suggest other similar appearing pathologies such as an annular pancreas.^10^

Radiological diagnosis of intestinal malrotation typically involves identifying abnormal positioning of the DJ junction during UGI fluoroscopy.^6^ In a normal scenario, the DJ junction is situated lateral to the left pedicle of the vertebral body adjacent to the duodenal bulb.^11^ Further UGI findings suggestive of malrotation or volvulus include: jejunum located on the right; duodenal redundancy and DJ junction corkscrew appearance of the distal duodenum and the proximal jejunum as the bowel twists around the mesentery.^7,12^ Demonstration of any of these findings usually leads to surgical exploration.^13^

In this case, the DJ junction was not noted in the expected position to the left of the left-sided pedicle of the vertebral body. Furthermore, the jejunum was noted to be lying on both sides of the abdomen. Keeping in mind that the patient was rotated, this is not typical for malrotation (Figure 1a). This may be due to the postero-superior course of the first segment of the duodenum. It is further believed that this abnormal presentation could in part be described by the presence of CPE.

Congenital peritoneal encapsulation is an extremely rare congenital malformation in which the small bowel is partially or totally surrounded by an accessory membrane.^14^ In a recent systematic review of the literature, 43 case studies were described, and none in the age group < 10 years old.^15^ A separate systematic review listed no reported cases from South Africa.^14^ The cause of CPE is not well understood but is thought to occur when the foetal mid-gut herniates into the umbilical cord at 8–10 week gestation, so understandably may be associated with malrotation.^14,16^

Usually, CPE remains asymptomatic and is an incidental finding during surgery, often to manage intestinal obstruction. It is uncertain whether the CPE in the case described was a contributor to the bowel obstruction or merely an incidental finding, due to the multiple pathologies involved. However, it would likely have been a contributor to the abnormal positioning of the duodenum on the fluoroscopy images that led to the diagnostic questioning.

Other rare entities causing small bowel encapsulation, like abdominal cocoon and sclerosing encapsulated peritonitis, often resemble CPE despite being distinct pathologies.^15^ These are differentiated by clinical history and presentation, as radiological imaging is typically inconclusive, with routine radiography or ultrasonography often showing no pathology.

While UGI series boasts a reported sensitivity of up to 100% for malrotation, it diminishes to 54% for mid-gut volvulus.^17^ Furthermore, imaging features can be ambiguous in up to 15% of cases, potentially leading to false positives or negatives.^6^ The use of additional modalities may enhance diagnostic confidence and accuracy. Small bowel follow-through to depict the location of the caecum may be added, as the caecum is abnormally positioned in 80% of patients with malrotation.^11^ As an alternative, emergency contrast enema may demonstrate an abnormal position of the colon.^6^ If deemed necessary, the treating surgeon should always be consulted because of the urgent nature of these presentations.

Effective collaboration between healthcare professionals, including radiologists, paediatric surgeons and other specialists, is essential for achieving accurate diagnoses and ensuring optimal patient outcomes. In this context, the seamless exchange of information, insights and expertise among team members facilitates a more thorough understanding of complex cases and enables informed decision-making regarding patient care.

## Conclusion

The study focuses on a unique presentation of bowel obstruction detected through fluoroscopy with a rare pathology. It offers insights into associated diagnostic challenges and underscores the value of utilising fluoroscopy in diagnosing complex gastrointestinal conditions. Furthermore, it highlights the significance of the crucial interdisciplinary collaboration between radiology and paediatric surgery in optimising patient care and outcomes.

The occurrence of bilious vomiting in neonates necessitates immediate investigation as it can swiftly escalate into a surgical emergency. A delay in diagnosis, particularly in cases of mid-gut volvulus, poses a risk of intestinal necrosis and increased mortality rates. Therefore, prompt recognition and intervention are imperative.

Being a rare cause, CPE is often overlooked as a potential differential diagnosis for abdominal pain and subacute small bowel obstruction.^15^ While it remains underdiagnosed, compounded by the difficulty in identification through imaging, treated patients have shown excellent postoperative recovery.^15^

This case study underscores the inherent clinical complexities associated with diagnosing malrotation complicated by mid-gut volvulus and rare CPE.
